# Usefulness Assessment of Automated Strabismus Angle Measurements Using Innovative Strabiscan Device

**DOI:** 10.3390/jcm13041067

**Published:** 2024-02-13

**Authors:** Ewa Grudzińska, Magdalena Durajczyk, Marek Grudziński, Łukasz Marchewka, Monika Modrzejewska

**Affiliations:** 1 Second Chair and Department of Ophthalmology, Pomeranian Medical University in Szczecin, 70-111 Szczecin, Poland; ewagrudz@gmail.com (E.G.); magdalena.durajczyk@gmail.com (M.D.); 2 Faculty of Mechanical Engineering and Mechatronics, West Pomeranian University of Technology in Szczecin, 70-310 Szczecin, Poland; marek.grudzinski@zut.edu.pl (M.G.); lukasz.marchewka@zut.edu.pl (Ł.M.)

**Keywords:** strabismus diagnostic device, strabismus angle measurement, image analysis, eye tracking

## Abstract

Background: The variability of the obtained results of manual tests assessing the angle of strabismus depends on the experience, skills, and training of the examiner. The authors hope that this new measuring device will provide a more sensitive and repeatable method for detecting small strabismus angles compared to the gold standard—PCT. The purpose of this article is to present an innovative strabismus angle demonstration device, called Strabiscan, to provide automated measurements of eye deviation and to compare the obtained results of these measurements to the traditional manual method. Methods: For patients with manifest strabismic disease (*n* = 30) and a group of healthy subjects (*n* = 30), a detailed history was taken and routine ophthalmologic examinations were performed, including best-corrected distance visual acuity, assessment of refractive error using an autorefractometer after cycloplegia, biomicroscopic evaluation of the anterior segment of the eye and evaluation of the eye fundus by indirect ophthalmoscopy. Subsequently, each patient and healthy subject was subjected to a prismatic cover–uncover test using a manual method, after which the presence of strabismus was detected and its angle assessed using a Strabiscan demonstration device. Results: In the control group using the Strabiscan demonstration device, small-angle latent strabismus ≤ 3DP was diagnosed in 83% of patients, while >3DP was found in 13%. In contrast, using the prismatic cover–uncover test, latent strabismus ≤ 3DP was diagnosed in only 13% of patients, and latent strabismus with an angle > 3DP was found in 13% of patients. No statistically significant differences were noted in the measurements of strabismus angles made by the different methods. Conclusions: The Strabiscan demonstration device allows quick and accurate assessment of the strabismus angle. Compared to the prismatic cover–uncover test, it has a higher sensitivity for detecting low-angle latent strabismus. Measurements with the Strabiscan do not require the presence of additional assistants for the test.

## 1. Introduction

Strabismic disease is a misalignment of the eyeballs, covering a heterogeneous group of eye movement disorders characterized by permanent or temporary deviation [1,2,3,4]. Strabismus occurs in 4–6% of the population worldwide with little geographic variation, in both children and adults, with the same frequency in both women and men [3,5,6,7]. There are limited data available on the global incidence of strabismus annually. An estimated 5.65% of children in China [8], 0.80% of children in Singapore [9], 2.47% children in the United States of America [10] and 0.8% in Africa [11] have strabismus.

Findings have shown that strabismic disease occurs in 65% of children by the age of three and is one of the most common visual disorders in preschool children [12]. Proper measurement of the type and magnitude of eye deviation is essential for the proper diagnosis, observation, and treatment of strabismus [13,14].

Strabismus may be intermittent or constant. It can be defined by the direction of the deviating eye as esotropia (inturning deviation), exotropia (out-turning deviation) or, less commonly, hypertropia (upturning deviation), hypotropia (downturning deviation) and cyclotropia (rotatory deviation) [15].

Strabismus can also be a manifestation of neurodevelopmental disorders, congenital abnormalities of extraocular muscles or their innervation and craniofacial disorders [16]. Although strabismus is commonly recognized among children, adults can also develop strabismus secondary to a variety of conditions, including trauma, surgical procedures, thyroid dysfunction, cranial nerve palsies or other neurologic diseases. The four most common types of new-onset strabismus are paralytic, convergence insufficiency, small-angle hypertropia and divergence insufficiency [17]. The risk factors for the various types of strabismus are complex and likely to be a combination of genetic and environmental factors. Some of the risk factors include a family history of strabismus, preterm birth, a neonatal care unit stay and epilepsy [16]. Among them, the greatest risk factors for comitant strabismus are anisometropia, maternal inheritance and critical retinopathy of prematurity [18].

Undetected and untreated strabismic disease leads to varying degrees of reduced stereoscopic vision and the development of amblyopia in the eye where strabismus is consistently present. It can also lead to postural disorders and torticollis, accompanied by spinal pain, mainly in the cervical region, which can lead to further long-term orthopedic complications. The condition reduces quality of life and impairs daily functioning [19]. The results from studies suggest that strabismus is also associated with increased odds of mental illness in children, particularly ADHD and anxiety disorder [20].

There are a few treatment techniques, depending on the type and magnitude of deviation and the age of the patient. Usually, the first step in managing any child with strabismus is to fully correct the refractive error. Surgery with adjustable and nonadjustable sutures could provide successful motor alignment outcomes in the majority of cases [21]. With surgery, we use many surgical techniques, including weakening and strengthening procedures, as well as vector adjustment procedures. Another method is the use of botulinum toxin type A, which causes temporary paralysis of the extraocular muscle and can improve the strabismus. Sometimes it is used as an adjunct to surgical therapy [22]. The next method is prism correction, which can be considered as an initial management in patients with a wide range and variety of etiologies of ocular deviations, including larger and mixed strabismus [23]. In children, they are usually prescribed for deviations < 20 PD. Orthoptic exercises are commonly used to treat intermittent exotropia [22].

### 1.1. Traditional Methods Assessment

The basic manual methods for diagnosing strabismic disease are the Hirschberg test, the Krimsky test, the cover–uncover test and the prismatic cover–uncover test. The Hirschberg test assesses the parallel alignment of the eyeballs based on the location of reflections on the surface of the illuminated corneas. The Krimsky test is a modification of the Hirschberg test, adding prisms in the visual axis which allows quantitative measurement of the amount of deviation for both near and far [24]. Both tests are considered the gold standard for testing eye alignment in uncooperative individuals, i.e., infants or people with mental disabilities [25,26]. The results of both the Hirschberg and Krimsky tests depend on the subjective judgment of the examiner and are much less accurate than the other available strabismus angle tests, even when performed by experienced strabologists [27,28]. Currently, the gold standard for assessing eye deviation is the cover–uncover test and the prismatic cover–uncover test, in which prism bars are additionally used. These tests should be performed for both near and far. They are time-consuming, and differences in measurements made by other researchers can be as high as 10 DP [29,30].

Prism bars are commonly used in ophthalmology and orthoptic offices to determine the strength of the corrective prism values by performing a prismatic cover test (PCT). When assessing eye deviation to the distance, the patient fixes his or her gaze on an object at a distance of 6 m, which requires a large space in the office [31]. This test can be unreliable due to the fact that it requires the examiner to simultaneously cover the patient’s eye, hold the prism bar, visually observe eye movements and correct the patient’s posture. Another complication of the PCT examination is the detection of the moment of stopping eye movement, which is particularly difficult when there are numerous reflections in the refractive glasses and prism bars. This is most often related to the unsuitable positioning of the examination chair in relation to the room lighting, over which the examiner has little influence. The manual occluder does not completely block the light entering the eye, and part of the periphery of the field of view (FoV) may result in partial recovery of binocular fusion. The strabismus examination involves the cooperation of the patient, remaining in a stable and fixed position for a long time, and, in the case of complex vertical and horizontal or oblique strabismus or uncooperative patients, requires the help of additional persons for head stabilization or the handling of additional prism bars. The accuracy of the examination is significantly reduced for large deviation values due to the variable resolution of the prism bars. The interval between prism power values is 1 DP in the 1–10 DP range, then 2 DP in the 10–20 DP range, and as much as 5 DP at higher values. In the PCT test, the prism bars should be aligned perpendicular to the optical axis of the eye in all planes, which again requires the experience of the examiner and is not verified in any way. The bars have no indicators of correct alignment, and the greater the deviation, the greater the measurement error. The skill and experience of the examiner and such factors as the psychophysical condition of the examiner and the patient, the time of day or coexisting diseases such as developmental disability play a significant role [31]. The difficulty of the tests performed and the role of the examiner’s experience is evidenced by the development of a virtual reality strabismus angle-assessment training application, the use of which significantly improves the examiner’s accuracy and efficiency [32].

An optical–mechanical synoptophore is also used to measure strabismic angles [33]. This device consists of two rotating optical tubes, which are attached to the patient’s eyes and display two complementary static images. As a result of the appropriate positioning of the tubes by the examiner or the patient himself, it is possible to combine the images into one image. At the same time, as the tubes progressively rotate, the images are alternately blanked out, forcing the patient’s eyes to adjust. The optometrist evaluates the strabismic angle at far and near fixation distance, based on the visual assessment of the adjustment movements and the actual angles of rotation of the tubes (read from the scale or digital encoders). Synoptophores are complicated to use and require a great deal of experience from the examiner, and the results depend on the psychophysical condition and subjective vision of the patient. In addition, numerous reports have been made by doctors and optometrists that there are problems with the evaluation of the strabismic angles in distant vision, especially the overestimation of the angles in convergent strabismus and the underestimation of the angles in divergent strabismus.

Advanced autorefractometers have implemented strabismic angle measurement by measuring corneal reflectance on the pupil area (e.g., PlusOptix). In a simplified form, this measurement is carried out manually by optometrists using a point light source held at a distance from the patient’s head, called the Hirschberg test [34]. The location of the reflections allows only a preliminary diagnosis of the strabismus type and the deviation values.

### 1.2. Scientific Articles

Researchers around the world are working to develop new devices to diagnose strabismic disease more accurately and quickly [35,36,37,38,39,40,41,42,43]. Numerous scientific articles report methods for the estimation of strabismus angles based on the analysis of reflections on the pupil area, reflections from the cornea and deeper layers and those based on the shape of the pupil in camera images. 

However, in our opinion, these methods are unreliable because they do not take into account the real path of light from the observed object to the macula on the retina, often far from the ideal anatomy of the eye, even though the Kappa angle is estimated. Both methods can only provide a preliminary estimate of the diagnosis showing the presence and type of strabismus.

To the best of our knowledge, there are no existing means or devices functionally and technically similar to the solution presented in this paper within the current state of the art.

## 2. Materials and Methods

### 2.1. Device Information

**Figure 1:**
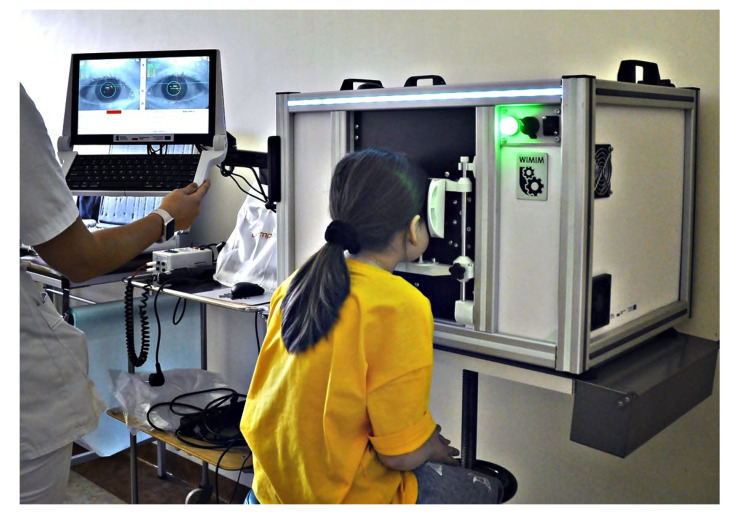
Strabiscan in the phase of pilot testing in clinical conditions involving pediatric patients.

Our proposed device, called Strabiscan, was developed in a technology demonstrator form at the 2nd Department of Ophthalmology of the Pomeranian Medical University, Szczecin, Poland (Figure 1). The device performs fully automatic measurements of strabismus angles with a resolution of 0.1 DP of the vertical and horizontal deviation of the eyeballs at near and far fixation. The Strabiscan device uses an advanced mathematical model of FoV and a very accurate eye-tracking system to determine strabismus angles, taking into account the individual characteristics of a patient, i.e., pupil distance, as well as distortions of the FoV caused by correction lenses. The device was developed as part of the Innovation Incubator 4.0 mini-grant, funded by the European Union.

### 2.2. Construction of the Device

**Figure 2:**
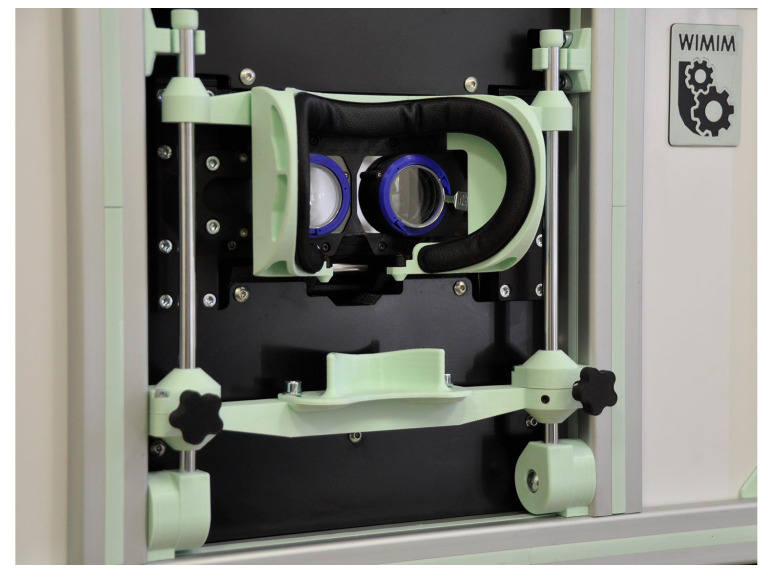
Patient’s head stabilization system.

The device, like many other ophthalmic devices, is stationary and integrated into a single housing. When seated, the patient rests his head and chin on a suitably contoured and permanently fixed frame (Figure 2). In addition, the design of the frame minimizes the amount of scattered ambient light falling onto the retina, making it easier to break up the binocular fusion. 

The pupil distance can be set from 53 to 82 mm and is taken into account when setting the following fixation point positions on the test screen, increasing the reliability of the results in both children and adults. The test screen was installed behind the optomechanical modules and in front of the patient’s eyes at a distance of 400 mm.

**Figure 3:**
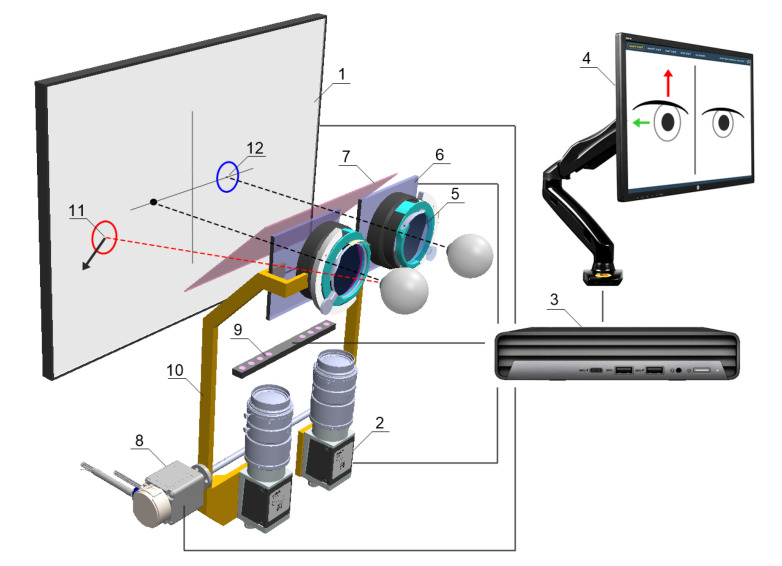
Schematic concept of the device: (1) fixation points display screen, (2) NIR camera, (3) PC, (4) operator control panel, (5) adapter for trial lenses, (6) LCD shutters, (7) hot mirror, (8) drive system allowing the optics to be adjusted to the user’s pupillary distance, (9) NIR LED illuminator, (10) movable fixing element for optical components, (11) fixation point for the non-fixing eye, (12) fixation point for the fixing eye.

The physical size of the screen workspace allows a large part of the patient’s FoV (70° horizontally and 40° vertically) to be covered during the examination. The patient, looking straight through the hot mirror, receives an image of the test screen in the visible band, while the image of the eye illuminated by the infrared LEDs is reflected by the mirror towards the cameras. A highly detailed image of the pupil is obtained, which enables an algorithm to detect adjusting movements of less than 0.2 mm, difficult to observe during a traditional PCT examination (Figure 3).

### 2.3. Fundamentals of Measurement Techniques

The device performs an automated measurement simulating PCT. During the measurement, alternating and cyclic LCD shutter switching is performed and the fixation point is displayed for the left or right eye exclusively, so that binocular vision is permanently switched off. In place of traditional prism bars that deflect the visual path, the device uses algorithms that simulate their operation and move the fixation point on the test screen surface. Patient cooperation is reduced to following and focusing the eye on the fixation point, which changes its position each cycle. The cameras capture and the computer analyses the intensity of the adjusting movements of the uncovered eyeballs. For the last position of the moving fixation point, the vertical and horizontal observation angles are determined. The device measures the total strabismus angle, encompassing both latent and manifest components.

### 2.4. Measurements

For the purpose of evaluating the usefulness of the device, 30 patients aged 5–52 years with manifest strabismic disease were tested with the Strabiscan device and with the prismatic cover–uncover test. The control group consisted of 30 healthy subjects (aged 5–25 years). The inclusion criteria for the study were manifest horizontal strabismus and age 5–75 years. On the other hand, exclusion criteria were mental or physical impairment disallowing cooperation during the test, age < 5 years, age > 75 years, refractive defect > ±10 D, other eye disease disabling fixation, systemic disease hindering the test, and low visual acuity (<0.1 Sn).

The study received a positive opinion from the Bioethics Committee at the Pomeranian Medical University (KB-0012/199/2020). The study protocol followed the tenets of the Declaration of Helsinki. Each study participant gave written permission to participate in the study.

A detailed history was taken for all patients, and routine ophthalmic examinations were performed, including best-corrected visual acuity to distance on Snellen charts (fixation points with numbers or pictures, depending on age). Each patient then underwent a prismatic cover–uncover test. The next test was the evaluation of the strabismus angle using the Strabiscan device. The final examinations were the evaluation of refractive error using an autorefractometer (Topcon KR-800, Tokyo, Japan, or Retinomax Righton) after cycloplegia with a drop of 1% cyclopentolate, biomicroscopic evaluation of the anterior segment of the eye, and evaluation of the fundus by indirect ophthalmoscopy using a Volk90D lens or with a Fison ophthalmoscope and a Volk20D lens.

The PCT test was conducted by two examiners: EG and MD—ophthalmology residents after pediatric ophthalmology training. The inter-operator differences never exceeded 5 DP. The procedure started with a 5 min one-eye occlusion. The test was conducted with the use of an opaque occluder.

In the proposed device, the appropriate set of the trial lens was installed and the patient, after stabilizing his head, was asked to observe the fixation point on the screen. The device calculated the trial lens for the far distance test according to the results entered into the system from the previous refraction measurement, adding a correction to compensate for the distance between the screen and the patient’s eyes (typically +2.5 D for a distance of 0.4 m). From the moment the head was placed on the device until the first test started, the patient could observe the background image monocularly on the screen. In this way, the breaking of binocular fusion was induced in the patient, crucial for relaxing the vision and obtaining a correct measurement result.

First, the device performed an automatic pupil–distance test, which involved the patient observing fixation points displayed sequentially exactly in front of the left eye and then in front of the right eye. During this time, the optical system adjusted its position so that the central point of the pupil was exactly halfway across the width of the images obtained from the cameras. Then, a strabismus angle test was performed at far fixation and then at near fixation distance, involving the patient’s observation of fixation points moving automatically on the screen in vertical and horizontal directions. During the alternating covering–uncovering of the eyes, the system was analyzing the adjusting movements of both eyes. For the near test, this correction was not applied. Three to five automatic measurements within one type of test were taken at a time, depending on the patient’s ability to cooperate with the device. A single measurement usually takes between 10 and 30 s. The operator has no control over when the measurement is completed.

During the test, the patient was asked to stay in a possibly stable position. Any slight movement of the patient’s head could have been interpreted by the device as the adjusting movement of the eye and could prevent an automatic completion of the test. This condition was particularly important in pediatric patients. Between measurements, the patient was also asked to keep his head in the device except during the replacing of trial lenses.

## 3. Results

Characterization of the collected patient features was carried out using descriptive statistics such as mean, standard deviation (SD), minimum and maximum values, as well as counts and percentages. Compliance with the normal distribution of continuous variables was checked with the W Shapiro–Wilk test. Comparisons of test values, separately at near and far fixation, between devices, and comparison of test annoyance between them, were made using the Mann–Whitney U test. A Receiver Operating Characteristic (ROC) curve was calculated based on the near and far examination results from each device, to compare the diagnostic capabilities of the devices. Parameters such as sensitivity, specificity and area under the ROC curve—AUC (area under the curve)—were calculated. In addition, the concordance of results from the two devices was compared using Cochran’s Q test. Results were considered statistically significant at *p* < 0.05.

Basic patient demographics are shown in Table 1. The study group included 30 patients with manifest strabismus, while the second group included 30 healthy subjects without manifest strabismus. The measurements taken were compared to the traditionally used alternating cover–uncover test using a prism bar, and a 55% concordance of positive results and a 45% concordance of negative results for distance were obtained. At near fixation, the percentage of positive results was concordant at 38.3% and negative at 61.7%. The device correctly detected strabismus in all subjects, as well as detecting small angles of hidden strabismus < 2 PD that were not detected by PCT (in 11 patients). The described difference is related to the difficulty of capturing, with the “naked” eye, the small adjusting movements of the eyes during the PCT test, which are easily detected by the Strabiscan device. Due to the described difficulties in the PCT test, we suggest that the values read from the Strabiscan device might be characterized by higher measurement accuracy. In the control group, using the Strabiscan device, small-angle latent strabismus ≤ 3 DP was diagnosed in 25/30 patients (83%) and >3 DP in 4/30 patients (13%). In contrast, using the PCT test, latent strabismus ≤ 3 DP was diagnosed in only 4/30 (13%) patients, and latent strabismus with an angle of >3 DP in 4/30 (13%) patients.

Strabismus at near fixation using the PCT test was diagnosed in 23 patients from the healthy group (76%) and in 28 patients with strabismic disease (93%). Using Strabiscan, strabismus at near fixation was observed in 28 patients from the healthy group (93%) and in 30 patients with strabismic disease (100%). Table 2 shows detailed features on the diagnosis and treatment of strabismic disease in the study group.

**Figure 4:**
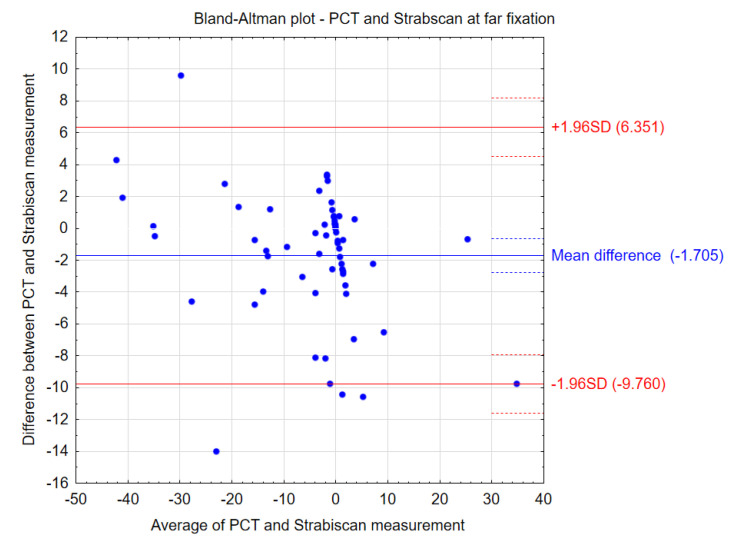
Bland–Altman plots showing the agreement between the compared procedures at far fixation.

**Figure 5:**
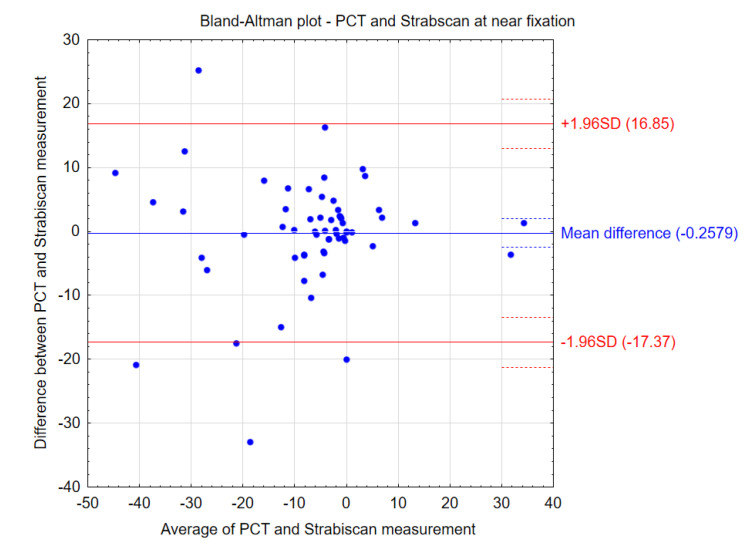
Bland–Altman plots showing the agreement between the compared procedures at near fixation.

The comparative results of the PCT method and the Strabiscan device are shown in Table 3. No statistically significant differences were noted in the measurements of strabismus angles performed by the different methods. Bland–Altman plots show the agreement between the compared procedures at far and near fixation (Figure 4 and Figure 5). Comparing the inconvenience of performing the tests for the patient, less inconvenience was noted in the test performed with the Strabiscan device than with the PCT method, but the difference was not statistically significant.

**Figure 6:**
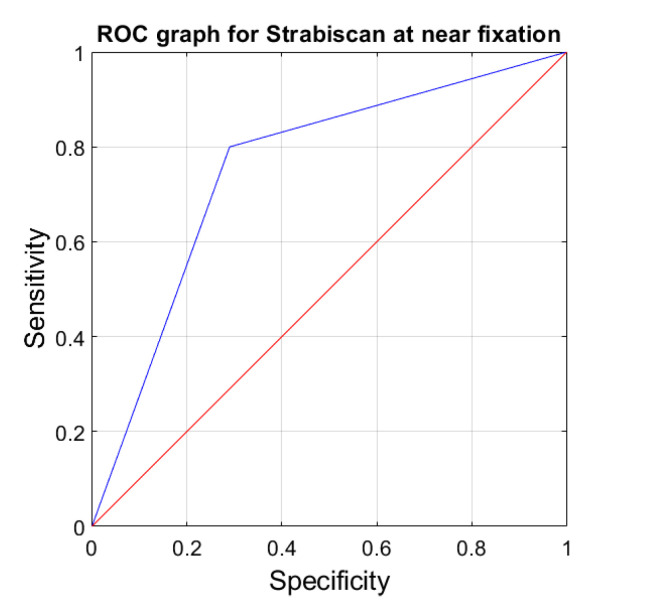
ROC graph for Strabiscan at near fixation. Sensitivity = 0.8; Specificity = 0.7; AUC = 0.75 (*p* < 0.001).

**Figure 7:**
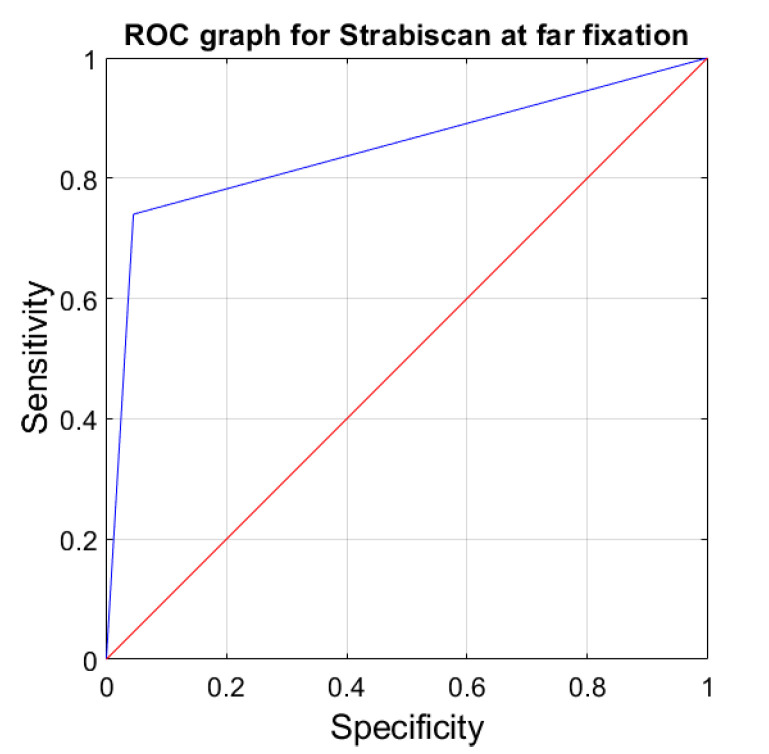
ROC graph for Strabiscan at far fixation. Sensitivity = 0.73; Specificity = 0.97; AUC = 0.85 (*p* < 0.001).

The sensitivity and specificity of strabismus detection with the Strabiscan device were analyzed. A Receiver Operating Characteristic (ROC) curve was calculated along with the area under the curve (AUC) (Figure 6 and Figure 7).

## 4. Discussion

Currently, there is no ideal method for measuring the strabismic angle, and although the prismatic cover test is the gold standard for the diagnosis of strabismic disease, it is known that the result depends on the examiner’s experience and subjective judgment. Obtaining precise results, independent of the experience and skills of the examiner, could lead to better results of surgery on the extraocular muscles, since measurements of the strabismus angle are not only the basis for the diagnosis of the type of strabismus, the degree of deviation of the eyeballs and the extent of this deviation, but also for the type and extent of surgery planned on the extraocular muscles.

In our study, we used an innovative piece of demonstration equipment called the Strabiscan. This device has no prism bars but a precisely adjusted optical system that takes into account refractive correction and the resulting prismatic effect, as well as fully controlled LCD shutters and fixation points displayed in positions tailored to the individual anatomical features of the patient, and therefore it does not have the disadvantages of the traditionally used methods and allows standardization of the examination in each patient. Better stabilization of the head on the device in relation to the PCT increases the accuracy and repeatability of the measurement. A constant measurement resolution of 0.1 DP is much greater in contrast to PCT, where the stepped resolution of prism power is available. In addition, the result of the study is not affected by the psychophysical state of the examiner, and the patient impact is limited due to the shorter examination time. The amount of deviation is observed by the software, not by the operator, so it cannot be affected by the individual operator’s experience level and perceptiveness. Another advantage of the Strabiscan device is the constant obscuration time of 1.5 s, and the covered eye is almost fully isolated from ambient light, allowing for more effective fusion breaking than in a standard PCT test using a simple manual occluder. This effect is comparable to the use of one-eye occlusion prior to PCT testing.

Strabiscan does not have the same limitation as other devices, which are based on eye movement, rely on the anatomical center of the pupil for positional assessment and do not contemplate the inclusion of the Kappa angle. Measurements conducted with our device have enhanced accuracy, as it catches the moment of stopping eye-adjusting movements while the eye is fixating.

The most debatable issue may be the accuracy of the measurements. The PCT test itself is the gold standard of testing, but there is no reference method for measuring thestrabismus angle to which new devices can be compared. As a new device, the Strabiscan does not have a specific measurement accuracy, but this will be the subject of future studies. In our opinion, the best indicator of the accuracy of the Strabiscan would be a study on a large group of patients conducted before and after surgery planned on the basis of the results from the PCT. The final postoperative result should then be confronted with the difference in strabismus angle readings between the Strabiscan and the PCT. Then, one would recalculate whether or not, hypothetically, a surgery performed according to the strabismus angle readings measured by the Strabiscan would also cover the postoperative strabismus angle.

The Strabiscan device successfully attempted to eliminate psychological convergence and the associated distance compression in a virtual environment by displaying a properly prepared and slightly blurred background image on the test screen, showing a landscape with far-field objects. Technological difficulties may be encountered if the patient has a very large refractive error, i.e., > ±10 Diopters (D), or if the patient has nystagmus. Underestimation of the strabismus angle may be encountered in the case of visual field deficits. 

A small technological risk is the imperfect pupil movement detection algorithm, which is based on very accurate detection of the pupil position in the image. The exact behavior of the algorithm in the case of very irregular pupils, or pupils strongly obstructed by the eyelid during oblique gaze, is not known. It should be emphasized that all the analyzed publications demonstrate eye examinations conducted under near-ideal conditions—i.e., the entire pupil is clearly visible, there are no shadows and the examinations are performed with the patient looking at a small angle. In contrast, the device we propose is designed to work effectively in contact with a variety of individual patients. Further research is needed to address these uncertainties.

Referring to the PCT method, we posit that the standard PCT test, which involves the patient wearing their habitual corrective lenses, yields incorrect readings from the prism bar. This is because, when looking at an angle, the border of the lens acts as a prismatic element, causing additional deflection of light rays. Our laboratory experiment demonstrated that, when looking at a fixation point set at a significant angle, there is a noticeable difference in the eyeball angle before and after the application of an additional corrective lens. For example, concave lenses give the patient an apparent angular narrowing of the FoV, which is generally the result of keeping the lens at a distance of about 10 mm from the front of the cornea. The PCT method does not determine the corrections of the viewing angles of the fixation point according to the size of the refractive error. Large values of astigmatism or aberrations of the corrective lenses further complicate the problem. This assertion necessitates further analysis of the results from strabismic angle measurements in patients wearing corrective lenses. The most pertinent findings may relate to the outcomes of strabismus surgeries that are based on traditional diagnostic methods. Particularly, it is important to investigate whether there is a statistically significant correlation between the type of refractive error and the degree of surgical undercorrection in strabismus. However, the prototype device lacks the ability to independently discern the latent and manifest components of strabismus. A primary limitation of the Strabiscan device is its requirement for a minimum interpupillary distance of 53 mm. Epidemiological studies indicate that the average pupillary distance of 53 mm corresponds to an age of approximately 4.8 years [44]. Moreover, the use of this device will be influenced by the child’s level of cooperation and concentration, which, in our opinion, will pose fewer challenges than with the PCT method. 

At the moment, the lack of devices such as Strabiscan on the market constitutes a strabismus measurement niche in public and private centers that treat strabismus with both behavioral and surgical methods. The proposed device is distinguished by its high reliability in strabismus angle assessment, and it may be easily used by ophthalmologists and orthoptists without specialized training courses, who, in turn, may encourage its widespread use in the healthcare system. In the current epidemiological situation related to COVID-19, an additional undoubted advantage of the device is the reduction in close contact with the patient and the possibility of using full personal protective equipment during examinations. Limitations of the current study include the small study group and the inclusion of Caucasians only. So far, results on only horizontal strabismus have been analyzed. It is not certain when the test can be performed on patients with severe mental disabilities.

## 5. Conclusions

In conclusion, Strabiscan is a valuable and safe demonstration device that enables quick and objective evaluation of the strabismus angle. As the first device of its kind in the world, it can automatically diagnose strabismus by performing a standard cover–uncover test, yielding results comparable to those obtained with the PCT test. Although the PCT is regarded as the gold standard, it has a significant number of disadvantages and, under certain conditions, its indications may be biased within a certain measurement error range. Compared to the prismatic cover–uncover test, it has a higher sensitivity for detecting low-angle latent strabismus. The Strabiscan device may enable accurate strabismus diagnosis even in ophthalmology offices that have not previously specialized in detecting and treating this condition, as its operation does not require highly qualified personnel. Furthermore, it could lead to improved outcomes in surgical treatment by providing accurate and reproducible measurements of strabismus angles.

## Figures and Tables

**Table 1:** Characteristics of the study groups.

	Study Group *n* = 30	Group of Healthy Subjects *n* = 30	*p*
Age [years]	16.6 ± 11.5	10.2 ± 3.8	0.002
Gender (F:M)	14: 16	17: 13	>0.05
Body height [m]	1.56 ± 0.19	1.41 ± 0.14	0.001
Body weight [kg]	53.98 ± 22.32	37.37 ± 12.55	0.001
Prematurity	4 (13.3%)	1 (3.3%)	>0.05
Burdened perinatal history	5 (16.7%)	0 (0.0%)	>0.05
Systemic diseases	9 (30%)	1 (3.3%)	0.03

**Table 2:** Features regarding diagnosis and treatment of strabismus in the study group.

Feature	Mean	SD	Min	Max
Age of diagnosis of strabismus [years]	3.35	3.11	0.00	12.00
Time since diagnosis of strabismus * [years]	13.33	13.24	1.00	52.00
Treatment time for strabismus ** [years]	10.80	11.78	1.00	45.00
	*n*	%		
Satisfaction with eyeglass treatment	17/30	60.7		
Willing to have faster strabismus surgery	17/30	60.7		
Use of orthoptic rehabilitation	24/30	80.0		
Satisfaction with orthoptic rehabilitation	18/30	69.2		
Undergoing eye surgery	15/30	50.0		
Strabismus surgery performed	14/30	46.7		
Strabismus in the family	9/30	30.0		
Ophthalmic disease in the family	8/30	26.7		

* Time since diagnosis of strabismus: for how long has the patient known he has strabismus? ** Treatment time for strabismus: for how long has the patient been treated for strabismus? (Conservative treatment with glasses, orthoptic exercises, as well as surgical treatment, qualified as treatment).

**Table 3:** Comparison of eye deviations and inconvenience of the examination for the patient performed with the listed methods.

	Mean ± SD	Min–Max	Median	Quartiles 25–75%	*p*
Eye deviation in the PCT test at far fixation [DP]	−5.9 ± 13.1	−40.0–30.0	0.0	−11.0–0.0	0.218
Eye deviation in the Strabiscan test at far fixation [DP]	−4.2 ± 14.3	−44.3–39.8	−0.6	−10.4–2.2	
Eye deviation in the PCT test at near fixation [DP]	−7.2 ± 14.9	−51.0–35.0	−4.0	−12.0–0.0	0.923
Eye deviation in the Strabiscan test at near fixation [DP]	−6.9 ± 14.6	−49.1–33.8	−3.9	−11.4–1.2	
Inconvenience of examination PCT	2.28 ± 1.95	1.00–10.00	1.00	1.00–3.00	0.531
Inconvenience of examination Strabiscan	2.04 ± 1.82	1.00–8.00	1.00	1.00–2.00	

## Data Availability

Dataset available on request from the authors.
